# Factors associated with employment and expected work retention among persons with multiple sclerosis: findings of a cross-sectional citizen science study

**DOI:** 10.1007/s00415-020-09973-3

**Published:** 2020-06-11

**Authors:** Anja I. Lehmann, Stephanie Rodgers, Christian P. Kamm, Mathias Mettler, Nina Steinemann, Vladeta Ajdacic-Gross, Marco Kaufmann, Jürg Kesselring, Pasquale Calabrese, Anke Salmen, Claudio Gobbi, Chiara Zecca, Georg F. Bauer, Viktor von Wyl

**Affiliations:** 1 Epidemiology, Biostatistics and Prevention Institute, University of Zurich, Hirschengraben 84, 8001 Zurich, Switzerland; 2 Department of Neurology, Inselspital, University Hospital Bern and University of Bern, Bern, Switzerland; 3 Neurocentre, Luzerner Kantonsspital, Lucerne, Switzerland; 4 Citizen scientist, 8422 Zurich, Switzerland; 5 Department of Neurology and Neurorehabilitation, Rehabilitation Centre Kliniken Valens, Valens, Switzerland; 6 Division of Molecular and Cognitive Neuroscience, University of Basel, Basel, Switzerland; 7 Department of Neurology, Multiple Sclerosis Center (MSC), Neurocenter of Southern Switzerland, 6900 Lugano, Switzerland; 8 Faculty of Biomedical Sciences, Università Della Svizzera Italiana (USI), 6900 Lugano, Switzerland

**Keywords:** Multiple sclerosis, Workplace, Psychosocial work conditions, Cross-sectional study

## Abstract

**Background:**

Multiple sclerosis (MS) notably affects adults of working age. For persons with MS (PwMS), being employed enhances their quality of life and it may be regarded as an indicator of overall functioning. Thus, ensuring work participation in PwMS is of general public health interest.

**Objective:**

To examine relevant socio-demographic, MS-, health- and work-related factors, including psychosocial working conditions, associated with currently working PwMS in Switzerland and their expected work retention.

**Methods:**

Using cross-sectional data of PwMS in the Swiss MS Registry (*n* = 541, median age = 48 [IQR 40;55]), multivariable logistic regression models were computed. First, currently working PwMS were characterised in comparison with those not currently working. Second, expected work retention, operationalized as subjective judgement “likely to work in the same job in 2 years”, was examined within the group of currently working PwMS.

**Results:**

The factors age (OR 0.96, 95% CI 0.92–0.99), sex (OR 0.28, 95% CI 0.13–0.60), highest achieved job position (OR 1.21, 95% CI 1.01–1.46), health-related quality of life (HRQoL) (OR 1.02, 95% CI 1.01–1.04) and the number of MS symptoms (OR 0.90, 95% CI 0.82–0.98) were associated with currently working PwMS. Moreover, HRQoL (OR 1.07, 95% CI 1.04–1.10) and psychosocial working conditions, such as job resources (e.g. autonomy, control or social support) (OR 2.83, 95% CI 1.50–5.33) and job demands (e.g. workload, time pressure) (OR 0.41, 95% CI 0.18–0.90) were important factors for expected work retention among this group.

**Conclusions:**

Resourceful psychosocial working conditions are crucial for PwMS to maintain employment. Employers could contribute to work retention among PwMS by creating a work environment with resourceful psychosocial working conditions and providing, for instance, social support.

**Electronic supplementary material:**

The online version of this article (10.1007/s00415-020-09973-3) contains supplementary material, which is available to authorized users.

## Introduction

Multiple sclerosis (MS) is a chronic disease of the central nervous system (CNS) with increasing prevalence estimates over the past 3 decades [1]. As MS commonly first occurs between 20 and 40 years of age, this disorder tends to affect adults of working age [1, 2]. Considering the range of cognitive, physical, and psychosocial impairments, MS can have severe consequences on a person’s ability to remain in the workforce [3–5]. However, for persons with MS (PwMS), work participation has a positive impact on social integration, self-esteem and health-related quality of life (HRQoL), not to mention financial security [6], and it may be regarded as an indicator of overall functioning [7]. Beyond this individual aspect, paid employment is also valuable to society [3]. Therefore, ensuring work participation among PwMS is of general public health interest.

International empirical evidence showed employment rates of PwMS ranging between 35.8 and 51.6% [2, 8, 9]. In a cross-sectional Swiss survey, 65% of PwMS below retirement age were employed [10]. However, nearly half of the PwMS working at the time of diagnosis reduced or left the workforce prematurely [3] because of disease- and work-related stressors [2, 11]. To prevent employee turnover, a better understanding of risk and protective factors associated with work participation and retention among PwMS is, therefore, highly important. Indeed, international research conducted in 20 European countries, the United States, Canada, Australia, and based on data from the world’s largest patient-driven MS registry NARCOMS [e.g. 3, 10, 12, 13–18], highlighted the importance of socio-demographic, MS- and (mental) health-related factors in relation to work [2, 6, 10]. Beyond socio-demographic characteristics, such as sex, age and education level, the following MS-related clinical indicators were judged to be relevant regarding work-related difficulties: the Expanded Disability Status Scale (EDSS), MS duration, a progressive MS course and certain MS-associated symptoms, such as fatigue, gait problems and cognitive and neuropsychological impairments [2, 10]. A recent meta-analytic review on psychological factors showed that depression, anxiety and certain coping abilities have a debilitating effect on employment [6].

These abovementioned studies have provided valuable insights into risk and protective factors associated with work participation and retention. Nonetheless, scant knowledge is available on the impact of the broad range of socio-demographic, MS- and health-related factors including contextual work-related factors [2, 9]. This is the first study to address this topic in Switzerland to date. Contextual work-related factors have been shown to play an important role among PwMS [3, 19–21], persons with chronic diseases as well as amongst the general population [22]. Specific contextual work-related factors, also referred to as psychosocial working conditions, are theoretically embedded in the job demands-resources [JD-R] model [23]. Job resources, such as autonomy, control or social support [23], are defined as the organisational, psychological, physical and social aspects of the job leading to attainment of work goals, development, and personal growth as well as a reduction in psychological or physiological costs and are considered to have a positive impact on well-being [23]. In contrast, job demands requiring physical and mental efforts refer to work characteristics linked to reduced health, namely the exhaustion component of burnout [24]. Job resources potentially crucial for work participation have been investigated, in general, for chronic diseases [22], but not specifically for MS, although each chronic disease has its own characteristics leading to disease-specific imponderabilities within the workplace.

Therefore, the aim of this study was to exploratively consider socio-demographic, MS-, health- and work-related factors (including the psychosocial working conditions: job resources and job demands) associated with currently working PwMS in Switzerland and their expected work retention. The research questions were (1) which factors characterise currently working compared to non-working PwMS in Switzerland, and (2) of the currently working PwMS, which factors are associated with expected work retention? The Swiss Multiple Sclerosis Registry (SMSR) provides a valid data set to address these questions. These findings may provide a basis for potential intervention strategies.

## Materials and methods

### Study sample

**Fig. 1:**
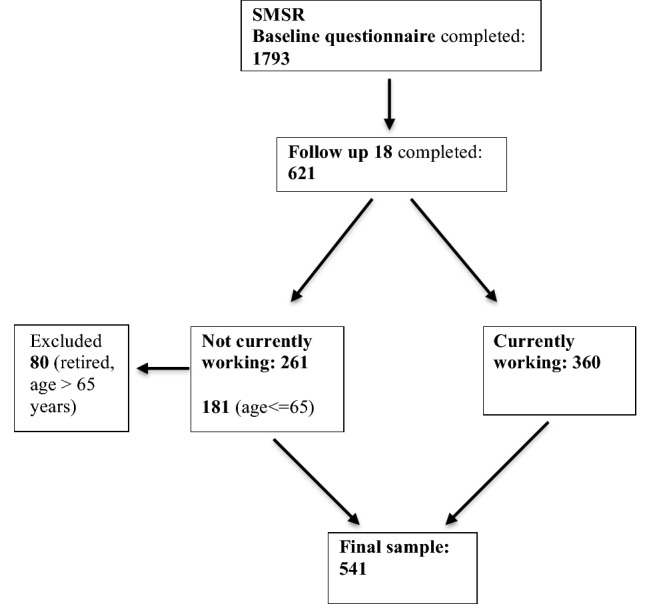
Flowchart describing the subsample composition of working versus not working persons in the Swiss Multiple Sclerosis Registry (numbers reflect numbers of persons)

The SMSR is an ongoing, nationwide self-reported registry for PwMS in Switzerland (*n* = 2277; status quo: April 06, 2020), initiated by and conducted in close collaboration with the Swiss Multiple Sclerosis Society (SMSS) (https://www.Clinical-Trials.gov identifier: NCT02980640) [25, 26]. This prospective, longitudinal, observational study is based on a citizen-science approach with PwMS representing the core element by being actively involved in relevant aspects, e.g. development of questionnaires and discussion of research findings. Details on the study design are described elsewhere [25, 26]. The SMSR was approved by the Ethics Committee of the Canton of Zurich (PB-2016-00894; BASEC-NR 2019-01027) and the participants signed a written informed consent after being informed about study procedure and aims in writing. For this study focusing on working versus not working PwMS, all data were extracted from a follow-up survey specifically addressing the topic MS and work (*n* = 621) (apart from information on socio-demographics implemented in the SMSR baseline assessment survey). Among the not currently working PwMS, retired persons (> 65 years; *n* = 80) were excluded leading to an overall age range between 21 and 67 years (some persons > 65 were still employed). The detailed subsample composition for the subsequent data analysis is depicted in Fig. 1.

### Measurements

#### Socio-demographic, MS- and health-related factors

The following categorical socio-demographic variables were dichotomised: education level (‘high’ = ‘high school’ [corresponding to 12–13 years of education], ‘higher professional education’ and ‘university/applied university’ versus ‘low’ = ‘school not finished, ‘mandatory school’, ‘apprenticeship'), civilian status (‘married, registered partnership’ versus ‘other’), living situation (‘living alone, single parenting’ versus ‘living with parents, spouse, friends’), and having children (‘yes’ if at least one child versus ‘no’). Scaling of the remaining socio-demographic variables was metric (age), binary coded (sex), and ordinal (highest achieved job position: ‘Labourers’ = ‘0’, ‘Machine operator’ = ‘1’, ‘Craft/Trade’ = ‘2’, ‘Agriculture’ = ‘3’, ‘Service’ = ‘4’, ‘Office/Admin’ = ‘5’, ‘Technician/Similar in different field’ = ‘6’, ‘Scientist/Teacher/Academic/Engineer’ = ‘7’, ‘Manager/Leading position’ = ‘8’), respectively.

The following categorical MS-related information was dichotomized: MS types (‘relapsing–remitting MS [RRMS]’ versus ‘progressive MS [PMS]’) and current disease-modifying treatment (DMT) (‘yes’ versus ‘no’). A sum score was computed including the current MS symptoms: vision, language, dysphagia, weakness, paralysis, fatigue, paresthesia, dizziness, pain, gait, balance, bladder, spasms, tics, tremor, gastrointestinal, epilepsy, sexual, memory, depression, concentration as well as spatial orientation and affective instability. Information on time since MS diagnosis was computed by subtracting the date of MS diagnosis from the date of the completed follow-up questionnaire.

HRQoL was measured by the European Quality of Life 5-Dimension 5-Level version (EQ-5D-5L). This instrument covers the following five dimensions: (1) mobility, (2) self-care, (3) usual activities, (4) pain/discomfort and (5) anxiety/depression [27, 28]. In addition, it provides an estimation of a single utility figure (also referred to as the EQ-5D-index) and a visual analogue scale (EQ-VAS). The French value set was used for the present study [29, 30] and the EQ-5D-index was rescaled from 0 (worst health) to 100 (best health) [31].

#### Work-related factors

The follow-up survey used for this study included the following self-report questionnaires: The shortened Multiple Sclerosis Work Difficulties Questionnaire (MSWDQ-23) [32], a short-version of the indicator tool developed by the United Kingdom Health and Safety Executive (HSE) [33], the Job Crafting Questionnaire (JCQ) [34], and the seven-item short version of self-endangering work behaviour [35]. For all these questionnaires, validated translations in German, French/Italian were applied if available or professionally translated by native speakers after obtaining permission from the original authors.

##### MS-related factors at work

The MSWDQ-23 provides information on work difficulties in PwMS. Apart from a total score, this instrument enables computation of the following three subscales: psychological/cognitive barriers, physical barriers and external barriers [32]. All items were measured on a five-point Likert scale ranging from 1 (“rarely/never”) to 5 (“almost always”). Besides the MSWDQ-23, we also asked the respondents about eight MS symptoms affecting their current work: pain, spasms, weakness, movement, balance, motoric skills, bladder and gastrointestinal tract (no = 0, yes = 1). A sum score was computed regarding these symptoms. Moreover, one item assessed whether the respondents had to change jobs because of MS (0 = no, 1 = yes).

##### Health-related behaviour at work

General health-related behaviour at work was measured using two scales: the JCQ [34] and the self-endangering work-behaviour scale [35]. The JCQ is divided into four independent job-crafting dimensions: (1) increasing social job resources, (2) increasing structural job resources, (3) increasing challenging job demands, and (4) decreasing hindering job demands. Previous research on the general population illustrated health-promoting mechanisms of job crafting [36]. For the present study, only the two items “I ask my supervisor to coach me” and “I ask colleagues for advice” were selected from the first dimension. All items were measured on a five-point Likert scale ranging from 1 (“rarely/never”) to 5 (“very often”).

The short version of the self-endangering work behaviour scale [37] is measured on a scale of seven items, such as working during recreation, working despite being ill, and consumption of substances to enhance performance. All items started with the question “In the last three months, how often has it occurred that …”. One example item is “In the last three months, how often have you worked at a pace that you could not maintain in the long run?” [35]. All items were measured on a five-point Likert scale ranging from 1 (“rarely/never”) to 5 (“very often”).

##### Psychosocial working conditions: job resources and job demands

The HSE was used to assess the psychosocial working conditions. This is a useful tool indicating whether and in what domain stress-related problems in the workplace might occur [33]. The HSE can be conceptualised into two subscales: job-related resources and demands [e.g. 38]. To measure job resources we chose a selection of six items from the subscales *control* (e.g. “I have some say over the way I work.”), *role clarity* (e.g. “I am clear about the goals and objectives of my department.”), *support of colleagues* (e.g. “I get the help and support I need from colleagues.”), *support of supervisor* (e.g. “My line manager encourages me at work.”), and *change* (e.g. “When changes are made at work, I am clear how they will work out in practice.”). Moreover, we chose two additional items (“At work, you can develop your skills” and “This work is varied”) from the salutogenetic subjective work analysis (SALSA = SALutogenetische Subjektive Arbeitsanalyse) questionnaire [39, 40].

For measuring job demands, three items from the HSE subscale *quantitative demands* (e.g. “I have to neglect some tasks because I have too much to do.”) and *negative relationships* (e.g. “I am subject to personal harassment in the form of unkind words or behaviour in my company.”) were used. Moreover, one item from the subscale *qualitative demands* of the SALSA (e.g. “It happens that work is too difficult for me.”) was chosen.

Besides the scales capturing psychosocial working conditions in general, we also asked the respondents whether they receive MS-related support from their employer (one item). All items were measured on a five-point Likert scale ranging from 1 (“rarely/never”) to 5 (“very often”).

### Statistical analysis

Descriptive statistics including frequencies and percentages for categorical data as well as medians and the inter-quartile range (IQR) for metric data were computed for employed and non-employed PwMS. Analyses for associations between the independent and the dependent variables were conducted with correlation analyses. To keep the regression models parsimonious, the non-significant independent variables (*p* > 0.10), except common control variables used in previous research (such as sex and time since MS diagnosis [2, 31]), were excluded from the following regression analyses. To explore factors associated with currently working PwMS, multivariable logistic regression models were fitted using socio-demographic characteristics as independent variables and the dichotomized item “currently working” versus “not currently working” as dependent variable. To explore factors associated with expected work retention among PwMS, a hierarchical logistic regression analysis was performed using socio-demographic, MS-, health-, and work-related characteristics as independent variables and the dichotomized item “likely to work in the same job in 2 years” versus “not likely to work in the same job in 2 years” as the dependent variable. The hierarchical approach was chosen in order to assess whether work-related factors explain variance in the dependent variable after accounting for socio-demographics, MS- and health-related factors. The independent variables were entered in four blocks: (1) socio-demographic, MS- and health-related factors, (2) MS-related factors at work, (3) health-related behaviour at work, and (4) psychosocial working conditions. This regression analysis was computed with a backward selection of variables to include only variables that made a significant (*p* < 0.10) contribution. Moreover, we included the confounding variables from block 1 in all four steps because evidence shows that these factors are associated with work participation [2, 6, 10].

All statistical analysis were performed using the IBM SPSS Statistics for Macintosh, versions 23.0 [41] and 25.0 [41].

## Results

Table 1 illustrates the descriptive analysis of the final sample (*n* = 541) split into those “currently working” (*n* = 360) versus those “not currently working” (*n* = 181). The sample of working PwMS was characterised by lower median age, higher levels of education, higher achieved job positions, more married persons or persons in a registered partnership, and less parents. In addition, this group encompassed fewer persons with PMS, shorter times since MS diagnosis, more persons currently on DMT, and a lower sum score of MS symptoms. Finally, the group of working PwMS showed a higher HRQoL compared with non-working PwMS. In contrast, neither sex nor the living situation significantly differed between the two groups (Table 1). Besides the descriptive analysis in Table 1, comorbidities are illustrated in the supplement (“Suppl. Descriptives”).

**Table 1:** Descriptive analysis of the study sample of working and not working persons with MS

Variable	Not working (*n* = 181)	Working (*n* = 360)	*p* Value	Total (*n* = 541)
Sex				
Male	35	91	0.12	126
	19.30%	25.30%		23.30%
Female	146	268		414
	80.70%	74.70%		76.70%
Age (median [IQR])	54 (46;60)	46 (38;53)	0.00	48 (40;55)
Education level				
Low (mandatory school, apprenticeship)	98	136	0.00	234
	56.60%	39.20%		45.00%
High (high school, higher professional education, university)	75	211		286
	43.40%	60.80%		55.00%
Highest achieved job position				
Unskilled worker	3	2	0.02	5
	2.20%	0.60%		1.10%
Machine operator	1	0		1
	0.70%	0.00%		0.20%
Craft/trade	6	12		18
	4.30%	3.80%		4.00%
Agriculture	1	0		1
	0.70%	0.00%		0.20%
Service	29	49		78
	20.90%	15.60%		17.20%
Office/admin	42	69		111
	30.20%	21.90%		24.40%
Technician/similar in different field	12	30		42
	8.60%	9.50%		9.30%
Scientist/teacher/academic/engineer	21	64		85
	15.10%	20.30%		18.70%
Manager/leading position	24	89		113
	17.30%	28.30%		24.90%
Civilian status				
Other	65	167	0.02	232
	36.50%	47.40%		43.80%
Married, registered partnership	113	185		298
	63.50%	52.60%		56.20%
Having children				
No	57	185	0.00	242
	31.50%	51.40%		44.70%
Yes	124	175		299
	68.50%	48.60%		55.30%
Living situation				
Living alone/single parenting	37	79	0.77	116
	21.10%	22.30%		21.90%
Living with parents/spouse/friends	138	276		414
	78.90%	77.70%		78.10%
MS-type				
Progressive MS (PMS)	71	53	0.00	124
	43.00%	15.60%		24.60%
Relapsing–remitting MS (RRMS)	94	286		380
	57.00%	84.40%		75.40%
Time since MS diagnosis (median [IQR])	13.5 (8;20.75)	8 (4;14)	0.00	10 (5;16)
Current disease-modifying treatment (past 6 months)				
No	63	71	0.00	134
	36.80%	20.00%		25.50%
Yes	108	284		392
	63.20%	80.00%		74.50%
Current MS symptoms (past 6 months)				
No symptom—Yes	9	59	0.00	68
	6.80%	18.10%		14.80%
Symptom: vision—Yes	49	60	0.00	109
	35.30%	21.80%		26.30%
Symptom: language—Yes	28	30	0.01	58
	21.10%	11.10%		14.40%
Symptom: dysphagia—Yes	28	29	0.01	57
	21.10%	10.60%		14.00%
Symptom: weakness—Yes	111	111	0.00	222
	72.10%	39.60%		51.20%
Symptom: paralysis—Yes	32	36	0.01	68
	24.40%	13.20%		16.90%
Symptom: fatigue—Yes	150	213	0.00	363
	89.30%	73.40%		79.30%
Symptom: paresthesia—Yes	106	187	0.07	293
	74.60%	66.10%		68.90%
Symptom: dizziness—Yes	57	90	0.07	147
	41.00%	32.00%		35.00%
Symptom: pain—Yes	97	104	0.00	201
	66.00%	37.30%		47.20%
Symptom: gait—Yes	108	123	0.00	231
	69.70%	43.20%		52.50%
Symptom: balance—Yes	114	142	0.00	256
	71.30%	49.70%		57.40%
Symptom: bladder—Yes	90	96	0.00	186
	59.60%	34.80%		43.60%
Symptom: spasms—Yes	97	97	0.00	194
	66.00%	34.80%		45.50%
Symptom: tics—Yes	37	41	0.00	78
	27.60%	15.10%		19.30%
Symptom: tremor—Yes	36	52	0.08	88
	26.90%	19.10%		21.70%
Symptom: gastrointestinal—Yes	74	62	0.00	136
	51.70%	22.50%		32.50%
Symptom: epilepsy—Yes	1	3	0.78	4
	0.80%	1.10%		1.00%
Symptom: sexual—Yes	46	46	0.00	92
	33.80%	16.80%		22.50%
Symptom: memory—Yes	65	74	0.00	139
	45.50%	26.80%		33.20%
Symptom: depression—Yes	37	46	0.01	83
	27.40%	16.90%		20.40%
Symptom: concentration—Yes	84	109	0.00	193
	54.90%	38.90%		44.60%
Symptom: spatial—Yes	26	19	0.00	45
	20.00%	7.00%		11.30%
Symptom: affective—Yes	41	35	0.00	76
	30.10%	12.90%		18.70%
Symptom: other—Yes	2	6	0.69	8
	1.60%	2.20%		2.00%
MS symptoms (sum score) (median [IQR])	9 (6;12)	5 (3;9)	0.00	6 (4;10)
HRQoL^a^ (median [IQR])	51.25 (30.50;68.72)	80.3 (58.75;92.9)	0.00	69.5 (50.1;91)

*MS* multiple sclerosis, *IQR* interquartile range

^a^HRQoL: health-related quality of life; European Quality of Life 5-Dimension 5-Level version (EQ-5D-5L)

### Research question 1

The correlation analyses showed that most of the variables (excepting civilian status and sex) were significantly (*p* < 0.01) associated with the outcome (see supplementary “Suppl. Correlation (RQ1)” for the correlation table). Table 2 illustrates the results.

**Table 2:** Associations between socio-demographic, MS- and health-related variables and working status [working vs. not working (ref.)] of persons with MS according to multivariable logistic regression models

	*B*	SE	OR	95% CI	
Sex (0 = male, 1 = female)	− 1.29	0.40	**0.28**	0.13	0.60
Age (per 1 year increase)	− 0.04	0.02	**0.96**	0.92	0.99
Education level (0 = low, 1 = high)	− 0.18	0.32	0.84	0.45	1.56
Highest achieved job position (0 = labourer to 8 = manager)	0.19	0.09	**1.21**	1.01	1.46
Civilian status (0 = other, 1 = married/partnership)	− 0.22	0.31	0.80	0.44	1.46
Having children (0 = no, 1 = yes)	− 0.11	0.31	0.90	0.49	1.63
MS-type (0 = PMS, 1 = RRMS)	− 0.18	0.38	0.84	0.40	1.76
Time since MS diagnosis (per 1 year increase)	− 0.03	0.02	0.97	0.94	1.01
Current disease-modifying treatment (past 6 months) (0 = no, 1 = yes)	0.63	0.33	1.89	0.98	3.61
MS symptoms (sum score)	− 0.11	0.05	**0.90**	0.82	0.98
HRQoL	0.02	0.01	**1.02**	1.01	1.04
Constant	2.72	1.41	15.24		
R^2^_(Nagelkerke)_	0.35				

*p* < 0.05 values in bold

*ref.* reference, *OR* odds ratio, *CI* confidence interval, *MS* multiple sclerosis, *RRMS* relapsing–remitting MS, *PMS* progressive MS, *HRQoL* health-related quality of life

The results show that sex, age, highest achieved job position, the number of MS symptoms and HRQoL were significantly associated with the working status (*p* < 0.05). More precisely, female respondents (*p* = 0.001), older respondents (*p* = 0.02) and respondents with more MS symptoms (*p* = 0.02) were less likely to work. Moreover, PwMS with a higher achieved job position (*p* = 0.04), and respondents with a higher level of HRQoL (*p* = 0.003) were more likely to work. No other independent variables were statistically significant at the 0.95 level of confidence (Table 2).

### Research question 2

Intercorrelations of the variables are shown in the supplement “Suppl. Correlation (RQ2)”. The results from the hierarchical logistic regression analysis are presented sequentially in Table 3. In the first step, the confounding variables (sex, age, MS type, current DMT, HRQoL and the MS symptoms sum score) were considered in the model. The results showed that MS-related factors [current DMT (*p* = 0.04) and time since MS diagnosis (*p* = 0.04)] as well as HRQoL (*p* < 0.001) were significantly associated with expected work retention. In the second step, MS-related factors at work (the sum score of MS-related symptoms at work, MS-related difficulties at work and the dichotomous item whether it was necessary to change work because of MS) were added under consideration of the confounder variables (Model 2). Only MS-related difficulties at work (MSWDQ-23) (*p* < 0.001) were significantly associated with expected work retention. Moreover, current DMT became non-significant when MS-related factors at work were inserted in the model. The third step included health-related behaviour at work (job crafting and self-endangering work behaviour) (Model 3). Only self-endangering work behaviour was significantly associated with expected work retention (*p* = 0.04). Moreover, time since MS diagnosis became insignificant when entering step 3 into the model. The fourth step also covered psychosocial working conditions [job demands, job resources and the dichotomous item whether MS-related support was received from the employer (Model 4)]. Model 4 was considered to be the best-fitting model (*R*^2^_(Nagelkerke)_ = 0.54). This model indicated that a higher level of HRQoL (*p* < 0.001), together with psychosocial working conditions, such as job demands (*p* = 0.03) and job resources (*p* = 0.001) were significantly associated with expected work retention. Furthermore, MS-related difficulties at work and self-endangering work behaviour were no longer significant after adding the psychosocial working conditions to the model.

**Table 3:** Associations between socio-demographic, MS-, health- and work-related variables and expected work retention [“likely to work in the same job in 2 years “vs.” not likely to work in the same job in 2 years” (ref.)] of persons with MS according to hierarchical logistic regression models

	Step 1: Socio-demographics, MS- and health-related factors					Step 2: MS-related factors at work					Step 3: Health-related behaviour at work					Step 4: Psychosocial working conditions				
	B	SE	OR	95% CI		B	SE	OR	95% CI		B	SE	OR	95% CI		B	SE	OR	95% CI	
Sex (0 = male, 1 = female)	− 0.59	0.48	0.56	0.22	1.41	− 0.35	0.50	0.70	0.26	1.88	− 0.33	0.51	0.72	0.27	1.95	− 0.52	0.54	0.59	0.20	1.72
Age (per 1 year increase)	− 0.01	0.02	0.99	0.95	1.04	− 0.02	0.03	0.98	0.94	1.03	− 0.03	0.03	0.97	0.93	1.02	− 0.04	0.03	0.96	0.91	1.02
MS type (0 = PMS, 1 = RRMS)	− 0.58	0.56	0.56	0.19	1.67	− 0.35	0.59	0.70	0.22	2.25	− 0.33	0.59	0.72	0.23	2.31	− 0.70	0.64	0.50	0.14	1.75
Current disease-modifying treatment (past 6 months), (0 = no, 1 = yes)	0.94	0.46	**2.56**	1.05	6.24	0.79	0.48	2.20	0.86	5.62	0.77	0.48	2.15	0.84	5.54	0.93	0.52	2.52	0.91	7.02
HRQoL^a^	0.07	0.01	**1.07**	1.05	1.10	0.06	0.01	**1.06**	1.03	1.09	0.06	0.01	**1.06**	1.03	1.09	0.07	0.01	**1.07**	1.04	1.10
MS symptoms (sum score)	0.03	0.06	1.04	0.92	1.17	0.13	0.07	1.14	0.99	1.31	0.14	0.07	1.15	1.00	1.32	0.04	0.08	1.04	0.89	1.22
Time since MS diagnosis (per 1 year increase)	0.07	0.03	**1.07**	1.01	1.13	0.07	0.03	**1.07**	1.01	1.14	0.06	0.03	1.06	1.00	1.13	0.05	0.03	1.05	0.99	1.12
MSWDQ-23^b^						− 1.59	0.45	**0.20**	0.08	0.50	− 1.23	0.49	**0.29**	0.11	0.76	− 0.56	0.53	0.57	0.20	1.63
Self-endangering work behaviour^c^											− 0.67	0.32	**0.51**	0.27	0.96	− 0.29	0.39	0.75	0.35	1.62
Job resources ^d^																1.04	0.32	**2.83**	1.50	5.33
Job demands ^d^																− 0.90	0.40	**0.41**	0.18	0.90
*R*^2^_(Nagelkerke)_	0.37					0.43					0.45					0.54				
Delta *R*^2^						0.06					0.02					0.09				

*p* < 0.05 values in bold

*MS* multiple sclerosis, *RRMS* relapsing–remitting MS, *PMS* progressive MS, *HRQoL* health-related quality of life, *OR* odds ratio, *CI* confidence interval

^a^European quality of life 5-dimension 5-level version (EQ-5D-5L)

^b^Shortened multiple sclerosis work difficulties questionnaire (MSWDQ-23)

^c^Seven-item short version of self-endangering work-behaviour scale

^d^Short-version of the indicator tool developed by the United Kingdom Health and Safety Executive (HSE) and from the salutogenetic subjective work analysis (SALSA = SALutogenetische Subjektive Arbeitsanalyse) questionnaire

Since job resources illustrated the strongest effects and have not specifically been investigated for PwMS up to date, descriptives of the specific job resources items with regard to expected work retention were calculated to explore the relative importance of the specific aspects of job resources (see supplementary “Suppl. Descriptive HSE (RQ2)”. Over 50% of those who expected to stay at work agreed to all items. Moreover, over 80% agreed to the items “I am clear about the goals and objectives of my department”, “This work is varied”, and “I can rely on my line manager to help me out with a work problem.” In a further step, all single items of the job resources scale were entered into the model [see supplementary “Suppl. Regression (RQ2)”]. Among the job resources, only the item “I can rely on my line manager to help me out with a work problem.” was significantly associated with expected work retention (*p* = 0.02).

## Discussion

In this Swiss MS Registry-based study, we examined relevant factors associated with the current working status and expected work retention of PwMS. Our main finding concerns the impact of psychosocial working conditions on expected work retention positively influenced by job resources (e.g. autonomy, control or social support) and negatively influenced by job demands (e.g. workload, time pressure). The other main finding shows that currently working PwMS are characterised by a higher job position and a higher level of HRQoL, while persons with female sex, a higher age and more MS symptoms, respectively, were less likely to work.

The impact of several of these socio-demographic, MS-, and health-related factors are in accordance with previous studies. We showed that older and female PwMS were more likely to be unemployed. The finding regarding age replicated previous findings on this socio-demographic characteristic [2, 42, 43]. Age might simply present a proxy for MS-disease-related characteristics, as higher age is linked to a higher degree of disability, progressive course and longer disease duration in PwMS [42]. However, the significant relationship between age and working remained even after statistically controlling for time since the MS diagnosis. Therefore, as in persons without MS, age is an important factor for work-related problems, which may be explained by age-related stigmatisation and discrimination [44]. With regard to the sex differences found, previous research also reported that males were more likely to be employed compared to females [45, 46]. Difficulties in managing home and work demands and differing coping strategies between the sexes were discussed as possible explanations, but evidence is consistent although particularly social gender roles and the continual evolution of female positions in the workforce in the past decades require further evaluation [6, 47, 48]. Moreover, it is questionable whether the association of male sex with employment would remain stable when eliminating other potential, non-MS-related causes from the analysis (such as pregnancy) [48]. It is also relevant if the proportion of females describing themselves as homemakers is excluded—as in our analysis—or not: a found male-preponderance of unemployed PwMS was related to this condition [49]. In fact, such sex differences were not consistently replicated between different countries: studies based on a Northwestern US [48] and Eastern Slovakian sample [50], respectively, did not report any differences between males and females [48]. Obviously, there are various factors influencing the examination of sex and employment status, which need to be taken into account.

PwMS with a higher number of current MS symptoms were more likely to be unemployed in our investigation. Previous research also indicated that PwMS with severe, multiple MS symptoms associated with higher disability, were more frequently unemployed [43, 51], and the likelihood was almost 20 times more [52]. Therefore, MS-related symptom management—optimised through early, supported illness disclosure [53, 54]—constitutes a possible intervention associated with successful employment outcomes as shown by longitudinal data [6, 14].

The current investigation demonstrated that high levels of HRQoL and high job positions were associated with employment. The link between high levels of HRQoL and employment was not surprising and supports previous studies showing this association among PwMS [7, 55]. This finding corroborates the notion that being at work might be regarded as an indicator of overall functioning. Apart from the income earned, regular employment provides a structure to daily life and social interaction, which are direct or indirect reasons for the positive impact of employment on HRQoL [55, 56]. Conversely, there is a wide range of negative consequences by loss of paid work [43, 57]. Beyond that, our results might inversely confirm the debilitating effect of depression and anxiety on employment, which are captured as dimensions within the instrument EQ-5D-5L measuring HRQoL [6, 58]. With regard to job position, however, previous MS-related research did not report any important effects of this socio-demographic variable, but rather focused on education. Some studies highlighted the impact of a high education level as a protective factor for employment [2], while others found that a lower level of education was associated with unemployment [42]. The effect of education was not significantly associated with employment when controlling for job position in our results. A higher job position might have a more direct effect on employment of PwMS, even if it is related to high education. In fact, our finding is in line with research on the general population indicating that a higher job position is associated with beneficial organisational outcomes, such as higher work engagement or lower turnover intentions [e.g. 59]. Overall, the factors high job position, HRQoL and employment status are inter-correlated and the question concerning causality of these factors still remains open: a multicentre study showed that education also had a strong influence on HRQoL, at least after a recent MS diagnosis [60]. This was explained by a stronger awareness and better abilities to cope with MS [60]. Longitudinal research considering the aspect of causality is thus warranted.

However, this is the first study highlighting the prominent role of contextual, psychosocial working conditions, such as job resources and job demands, with regard to expected work retention in PwMS. Our results showed that when entering psychosocial working conditions to the model, MS-related difficulties and health-related behaviour at work, such as self-endangering work behaviour, were no longer significant. Up to now, such environmental work-related factors have not been sufficiently considered by previous studies—if at all—they have been investigated within chronic illnesses in general [22], but not specifically for PwMS.

HRQoL also had an important impact on expected work retention in the present study. This could be explained by the concept of self-efficacy which might mediate the relationship between physical functioning (as an indicator of HRQoL) and work instability [61]. Self-efficacious people are more confident and have better disease management skills. This means that PwMS who are functioning well might rather have internalised a confident attitude regarding their future work participation. However, because our results rely on cross-sectional data, causal conclusions are not possible. Based on our findings, a bi-directional relationship between HRQoL and work participation is conceivable: work participation supports overall functioning since it promotes recovery, social inclusion and citizenship, and people who are functioning well are more confident about remaining in the workforce in the long run.

### Practical implications

Our findings raise important questions concerning the practical implications for employers. How must the working environment be created so that PwMS can maintain their professional activity as long as possible?

On the one hand, previous studies provide information on the infrastructural circumstances: workplace accessibility and certain facilities, such as toilet access and sufficient resting possibilities, should be a given. Some of these aspects are in fact socio-political issues, as stated by Uccelli et al. [3]. On the contrary, flexible working hours are subjectively valuable working conditions for PwMS, which can be directly influenced by the employers. The adaption of working times depending on the current complaints is not only beneficial for the overall state of health but also enhances work-related efficiency. Also MS societies may play an important role in supporting PwMS by negotiating work changes, such as flexible hours and in educating employers in MS-related work topics [3].

On the other hand, the findings from the present study provide answers from a psychosocial perspective. In particular, the items behind the scale of job-related resources are instructive: employers should ensure working conditions provide employees with sufficient control (77% of those who intend to stay at work rather or totally agree on that item), that is to say, employees must be able to co-decide on the way they work. Moreover, employees should be clear on the concrete goals and objectives of their department (93% of those who intend to stay at work rather or totally agree on that item), which also depends on the employer. Beyond these aspects of control and role clarity, social support is an essential component to raise work-related resources. Colleagues should help and support PwMS whenever needed (74% of those who intend to stay at work rather or totally agree on that item). This social support is also required from the side of the superiors: line managers should encourage their employees at work (67% of those who intend to stay at work rather or totally agree on that item) and provide support when problems arise (81% of those who intend to stay at work rather or totally agree on that item). Moreover, PwMS should be able to develop their skills (67% of those who intend to stay at work rather or totally agree on that item). This development must be regularly adapted depending on the degree of MS-related disability. Moreover, PwMS should be clear about changes at work and how they will work out in practice (60% of PwMS who intend to stay at work rather or totally agree on that item) and work should be varied. Due to disease-related absences, PwMS might miss important information regarding organisational changes that could lead to confusion when returning to work. Therefore, they should be adequately informed about past organisational decisions.

When exploring the relative importance of these job resources items in an additional regression analysis [“Suppl. Regression (RQ2)”], we found only the item “I can rely on my line manager to help me out with a work problem.” was significantly associated with expected work retention. Therefore, managerial support appears to be *particularly* important. Nevertheless, it is also of note that 50% of those who expect to stay at work agreed to *all* job resources items. Therefore, one may also argue that all components of job resources and most likely their *interaction* are key to work participation. The basis of all these psychosocial components is a good communication between employers and employees. We, therefore, suggest a regular personal exchange to stay in touch with the needs of the employees. Finally, employers should be aware of the fact that the chances to detect such personal needs are significantly increased by a confiding relationship with their employees.

### Limitations and future research

This study stands out through notable strengths as it is based on the innovative study design of the SMSR enabling the enrolment of PwMS not usually included in samples derived from MS centres [26]. Nevertheless, the present study has some limitations to be considered when interpreting the results. These include the reliance on self-reported data. However, appraisals of the psychosocial working conditions, work and/or MS-related difficulties experienced are subjective and thus are difficult to assess with other methods than self-report data. Another limitation is the cross-sectional design that hinders causal conclusions. Moreover, potential moderators and mediators were not assessed to explain the complex mechanisms between MS and work participation. Future research could disentangle these complex influences of factors, for example, by path modelling. Furthermore, concerning the group of persons rating the probability of remaining in the same job in 2 years as low, we cannot exclude alternative explanations, such as changes of position within the same company, changes of contract (from employed to self-employed), or even due to upcoming retirement. Also, even though some aspects of comorbidities were captured by the considered EQ-5D-5L, more detailed comorbidities were only descriptively presented (“Suppl. Descriptives”) but not included in the regression model although they might be associated with working status or expected work retention. Moreover, we did not examine the impact of the different MS symptoms in the regression models. Although our descriptive data illustrated that the sample of working PwMS was characterised by a lower frequency of most of the MS symptoms, it is of note that fatigue was the most prominent symptom mentioned by the whole sample (both working and non-working PwMS). Previous research has underlined that fatigue is highly common in PwMS, is associated with high subjective burden [62] and beyond that demonstrated the important role of fatigue for employment loss [14]. Besides the resulting cognitive problems, fatigue is invisible to others which could make accommodations difficult [11]. Because the mechanisms of fatigue appear to be complex and multifaceted, the causal impact of fatigue and its interplay with work-related factors should be examined in future longitudinal research. Finally, it is important to underline that there might be several inter-personal differences not adequately reflected by our statistical approach. For example, we cannot exclude the possibility that in case the working conditions are suboptimal, unemployment might in fact reduce stress in certain individuals. Moreover, such unresolved heterogeneity is not only conceivable on the individual but also on the contextual level. The impact of our examined factors could depend on the specific professional activity. Accordingly, the influence of certain MS symptoms, such as gait disturbances, might differ if a person works in an office or on a construction site. Future analysis reducing such heterogeneity would be valuable for the persons concerned.

## Conclusion

This study highlights the important role of work participation among PwMS. Socio-demographics (age, sex, job position), HRQoL and MS-related factors such as number of symptoms were associated with currently working PwMS. Moreover, HRQoL and job resources were important factors for expected work retention among those currently working. Considering these findings, pursuing gainful employment is quite possible for PwMS, particularly through the support of employers. They could contribute to work retention among PwMS by shaping a work environment with resourceful psychosocial working conditions—social support is only one of several possibilities. With these findings, we believe that we are the first to demonstrate that resourceful psychosocial working conditions are not only crucial in the general population, but also for PwMS. Based on this knowledge, we plan to develop guidelines for PwMS and employers of PwMS.

## Electronic supplementary material

Below is the link to the electronic supplementary material.

Supplementary file1 (DOCX 18 kb)

Supplementary file2 (DOCX 14 kb)

Supplementary file3 (DOCX 19 kb)

Supplementary file4 (DOCX 17 kb)

Supplementary file5 (DOCX 19 kb)
