# Supplementary material for: Factors associated with employment and expected work retention among persons with multiple sclerosis: findings of a cross-sectional citizen science study
Source: J Neurol. 2020 Jun 11;267(10):3069–82. doi: 10.1007/s00415-020-09973-3 (PMC7501110; doi:10.1007/s00415-020-09973-3)
Supplement: Supplementary file 1 — Supplementary file1 (DOCX 18 kb) [file 415_2020_9973_MOESM1_ESM.docx]

Intercorrelations of the variables

|  |  | 2 | 3 | 4 | 5 | 6 | 7 | 8 | 9 | 10 | 11 | 12 | 13 | 14 | 15 | 16 | 17 | 18 | 19 | 20 |
| --- | --- | --- | --- | --- | --- | --- | --- | --- | --- | --- | --- | --- | --- | --- | --- | --- | --- | --- | --- | --- |
| 1 | Expected work retention | .02 | -.18^**^ | .08 | .04 | -.06 | -.06 | .01 | .22^**^ | -.02 | .13^**^ | -.32^**^ | .52^**^ | -.40^**^ | -.53^**^ | .16^**^ | .32^**^ | -.26^**^ | -.15^**^ | -.29^**^ |
| 2 | Sex (0 = male, 1 = female) |  | -.21** | -.17** | -.24^**^ | -.13** | -.10* | -.08 | .22** | -.07 | .16** | -.22** | .17** | -.16** | -.02 | -.03 | -.04 | .07 | .06 | .02 |
| 3 | Age (per 1 year increase) |  |  | -.02 | .16** | .18** | .29** | -.09 | -.44** | .42** | -.18** | .25** | -.41** | .32** | .19** | .07 | -.03 | -.11* | -.08 | -.02 |
| 4 | Education level (0 = low, 1 = high) |  |  |  | .48** | .08 | -.04 | .14** | -.08 | .06 | .07 | -.02 | .10* | -.07 | -.16** | -.02 | .14** | -.11* | .06 | -.01 |
| 5 | Highest achieved job position (1 = labourer to 8 = manager) |  |  |  |  | .11* | -.01 | .10* | -.15** | .10* | -.07 | .09 | -.04 | .09* | -.02 | .07 | .15** | -.02 | .04 | .15** |
| 6 | Civilian status (0 = other, 1 = married/partnership) |  |  |  |  |  | .44** | .56** | -.06 | .07 | .01 | .07 | -.06 | .08 | .04 | .09 | .02 | -.10* | -.12* | -.06 |
| 7 | Having children (no = 0, yes = 1) |  |  |  |  |  |  | .17** | -.13** | .11* | -.08 | .07 | -.11* | .12** | .02 | .08 | .04 | -.11* | -.06 | -.10* |
| 8 | Living situation |  |  |  |  |  |  |  | .05 | -.04 | .02 | .02 | .05 | .01 | -.04 | .16** | .13** | -.11* | -.07 | -.04 |
| 9 | MS type (0 = PMS, 1 = RRMS) |  |  |  |  |  |  |  |  | -.32** | .21** | -.36** | .50** | -.35** | -.23** | -.12* | .00 | .02 | -.12* | -.05 |
| 10 | Time since MS diagnosis (per 1 year increase) |  |  |  |  |  |  |  |  |  | -.16** | .13* | -.24** | .17** | .07 | .12* | .05 | -.07 | .04 | -.07 |
| 11 | Current disease modifying treatment (0 = no, 1 = yes) |  |  |  |  |  |  |  |  |  |  | -.06 | .10* | -.14** | -.08 | -.08 | -.02 | .04 | -.03 | .00 |
| 12 | Sum score MS symptoms |  |  |  |  |  |  |  |  |  |  |  | -.71** | .59** | .60** | .08 | -.12* | .05 | .18** | .31** |
| 13 | HRQoL |  |  |  |  |  |  |  |  |  |  |  |  | -.69** | -.68** | -.12* | .15** | -.09* | -.21** | -.30** |
| 14 | Symptoms affecting work (sum) |  |  |  |  |  |  |  |  |  |  |  |  |  | .58** | .11* | -.14** | .09 | .16** | .32** |
| 15 | MSWDQ-23 |  |  |  |  |  |  |  |  |  |  |  |  |  |  | -.07 | -.34** | .37** | .23** | .52** |
| 16 | Receiving support from employer (0 = no, 1 = yes) |  |  |  |  |  |  |  |  |  |  |  |  |  |  |  | .38** | -.31** | .11* | -.15** |
| 17 | Job resources |  |  |  |  |  |  |  |  |  |  |  |  |  |  |  |  | -.31** | .06 | -.22** |
| 18 | Job demands |  |  |  |  |  |  |  |  |  |  |  |  |  |  |  |  |  | .23** | .53** |
| 19 | Job crafting (support) |  |  |  |  |  |  |  |  |  |  |  |  |  |  |  |  |  |  | .17^**^ |
| 20 | Self-endangering work behaviour |  |  |  |  |  |  |  |  |  |  |  |  |  |  |  |  |  |  |  |
|  | ***Note.*** * p < .05 (one-tailed), ** p < .01 (one-tailed)  Correlations with job positions were calculated with the Spearman correlation coefficient. All other variables were calculated with the Pearson correlation coefficient. | | | | | | | | | | | | | | | | | | | |

Abbreviations:

MS = Multiple Sclerosis

RRMS = Relapsing-remitting MS

PMS = Progressive MS

HRQoL: Health-related quality of life
