# Supplementary material for: Factors associated with employment and expected work retention among persons with multiple sclerosis: findings of a cross-sectional citizen science study
Source: J Neurol. 2020 Jun 11;267(10):3069–82. doi: 10.1007/s00415-020-09973-3 (PMC7501110; doi:10.1007/s00415-020-09973-3)
Supplement: Supplementary file 2 — Supplementary file2 (DOCX 14 kb) [file 415_2020_9973_MOESM2_ESM.docx]

Descriptives of the specific job resources items with regard to expected work retention

|  | Expected work retention | | | |
| --- | --- | --- | --- | --- |
|  | partly agree | | rather agree/totally agree | |
|  | N | % | N | % |
| Job resources: "I have some say over the way I work." | 45 | 15.60% | 221 | 76.50% |
| Job resources: "I am clear about the goals and objectives for my department." | 12 | 4.20% | 270 | 93.40% |
| Job resources: "At work, you can develop your skills." | 61 | 21.30% | 191 | 66.60% |
| Job resources: "This work is varied." | 35 | 12.10% | 240 | 83.00% |
| Job resources: "I get help and support I need from colleagues." | 42 | 14.70% | 212 | 74.40% |
| Job resources: "My line manager encourages me at work." | 49 | 17.30% | 189 | 66.50% |
| Job resources: "I can rely on my line manager to help me out with a work problem." | 30 | 10.60% | 230 | 81.10% |
| Job resources: "When changes are made at work, I am clear how they will work out in practice." | 57 | 20.10% | 170 | 59.90% |
