# Supplementary material for: Factors associated with employment and expected work retention among persons with multiple sclerosis: findings of a cross-sectional citizen science study
Source: J Neurol. 2020 Jun 11;267(10):3069–82. doi: 10.1007/s00415-020-09973-3 (PMC7501110; doi:10.1007/s00415-020-09973-3)
Supplement: Supplementary file 3 — Supplementary file3 (DOCX 19 kb) [file 415_2020_9973_MOESM3_ESM.docx]

Descriptive statistics of comorbidities

| **Comorbidities** |  | **Not Working  (n = 181)** | **Working  (n = 360)** | **Total  (n = 541)** |
| --- | --- | --- | --- | --- |
| No comorbidities | yes | 114 | 258 | 372 |
|  |  | 73.10% | 74.40% | 74.00% |
| Diabetes type 1 | yes | 1 | 0 | 1 |
|  |  | 2.30% | 0.00% | 0.80% |
| Diabetes type 2 | yes | 0 | 2 | 2 |
|  |  | 0.00% | 2.20% | 1.50% |
| Cancer | yes | 2 | 1 | 3 |
|  |  | 4.70% | 1.10% | 2.30% |
| High blood pressure | yes | 8 | 12 | 20 |
|  |  | 17.80% | 13.00% | 14.60% |
| Cardiac problems | yes | 5 | 4 | 9 |
|  |  | 11.60% | 4.50% | 6.80% |
| Depression | yes | 10 | 10 | 20 |
|  |  | 21.70% | 11.20% | 14.80% |
| Osteoporosis | yes | 4 | 4 | 8 |
|  |  | 9.10% | 4.40% | 6.00% |
| Rheumatic disorder | yes | 6 | 1 | 7 |
|  |  | 13.00% | 1.10% | 5.20% |
| Orthopedic | yes | 16 | 19 | 35 |
|  |  | 32.00% | 21.10% | 25.00% |
| Skin disease | yes | 7 | 4 | 11 |
|  |  | 15.90% | 4.50% | 8.30% |
| Psoriasis | yes | 4 | 2 | 6 |
|  |  | 9.30% | 2.20% | 4.50% |
| Asthma | yes | 1 | 0 | 1 |
|  |  | 2.40% | 0.00% | 0.80% |
| Other | yes | 15 | 28 | 43 |
|  |  | 32.60% | 30.40% | 31.20% |
