# Supplementary material for: Factors associated with employment and expected work retention among persons with multiple sclerosis: findings of a cross-sectional citizen science study
Source: J Neurol. 2020 Jun 11;267(10):3069–82. doi: 10.1007/s00415-020-09973-3 (PMC7501110; doi:10.1007/s00415-020-09973-3)
Supplement: Supplementary file 4 — Supplementary file4 (DOCX 17 kb) [file 415_2020_9973_MOESM4_ESM.docx]

Regression analysis with the specific job resources items

|  | B | SE | OR | 95%CI | |
| --- | --- | --- | --- | --- | --- |
| Sex (0 = male, 1 = female) | -0.55 | 0.59 | 0.58 | 0.18 | 1.85 |
| Age (per 1 year increase) | -0.04 | 0.03 | 0.96 | 0.91 | 1.02 |
| MS-type (0 = PMS, 1 = RRMS) | -0.62 | 0.65 | 0.54 | 0.15 | 1.93 |
| Current disease modifying treatment (0 = no, 1 = yes) | 1.15 | 0.54 | **3.15** | 1.10 | 9.05 |
| HRQoL | 0.06 | 0.01 | **1.06** | 1.03 | 1.09 |
| MS symptoms (sum score) | 0.03 | 0.08 | 1.03 | 0.87 | 1.21 |
| Time since MS diagnosis (per 1 year increase) | 0.05 | 0.03 | 1.05 | 0.99 | 1.12 |
| MSWDQ-23 | -0.73 | 0.55 | 0.48 | 0.16 | 1.42 |
| Self-endangering work behaviour | -0.24 | 0.40 | 0.78 | 0.36 | 1.72 |
| Job demands | -0.89 | 0.42 | **0.41** | 0.18 | 0.94 |
| Job resources: "I have some say over the way I work." | 0.20 | 0.25 | 1.22 | 0.75 | 2.00 |
| Job resources: "I am clear about the goals and objectives for my department." | -0.23 | 0.32 | 0.80 | 0.43 | 1.49 |
| Job resources: "At work, you can develop your skills." | 0.00 | 0.27 | 1.00 | 0.58 | 1.71 |
| Job resources: "This work is varied." | 0.30 | 0.32 | 1.35 | 0.71 | 2.54 |
| Job resources: "I get help and support I need from colleagues." | 0.03 | 0.22 | 1.03 | 0.66 | 1.60 |
| Job resources: "I can rely on my line manager to help me out with a work problem." | 0.47 | 0.20 | **1.59** | 1.07 | 2.38 |
| Job resources: "When changes are made at work, I am clear how they will work out in practice." | 0.33 | 0.19 | 1.40 | 0.97 | 2.01 |
| R² _(Nagelkerke)_ | 0.57 |  |  |  |  |
| ***Note.*** The item "My line manager encourages me at work." was not included in the analyses due to multicollinearity. | |  |  |  |  |

Abbreviations:

MS = Multiple Sclerosis

RRMS = Relapsing-remitting MS

PMS = Progressive MS

HRQoL: Health-related quality of life

OR = Odds ratio; CI = Confidence interval

p< 0.05 values in bold
