# Supplementary material for: Factors associated with employment and expected work retention among persons with multiple sclerosis: findings of a cross-sectional citizen science study
Source: J Neurol. 2020 Jun 11;267(10):3069–82. doi: 10.1007/s00415-020-09973-3 (PMC7501110; doi:10.1007/s00415-020-09973-3)
Supplement: Supplementary file 5 — Supplementary file5 (DOCX 19 kb) [file 415_2020_9973_MOESM5_ESM.docx]

Intercorrelations of the variables

|  |  |  |  |  |  |  |  |  |  |  |  |  |  |
| --- | --- | --- | --- | --- | --- | --- | --- | --- | --- | --- | --- | --- | --- |
|  |  | 2 | 3 | 4 | 5 | 6 | 7 | 8 | 9 | 10 | 11 | 12 | 13 |
| 1 | Working | -.07 | -.33^**^ | .17^**^ | .16^**^ | -.10^**^ | -.19^**^ | -.01 | .30^**^ | -.30^**^ | .18^**^ | -.32^**^ | .43^**^ |
| 2 | Sex (0 = male, 1 = female) |  | -.15^**^ | -.16^**^ | -.21^**^ | -.07^*^ | -.05 | -.05 | .18^**^ | .00 | .12^**^ | -.08^*^ | .09^*^ |
| 3 | Age (per 1 year increase) |  |  | -.10^**^ | .08^*^ | .19^**^ | .30^**^ | -.03 | -.44^**^ | .45^**^ | -.23^**^ | .24^**^ | -.40^**^ |
| 4 | Education level (0 = low, 1 = high) |  |  |  | .50^**^ | .01 | -.05 | .08^*^ | .02 | -.06 | .04 | -.08^*^ | .18^**^ |
| 5 | Highest achieved job position (0 = labourer to 8 = manager) |  |  |  |  | .07 | -.06 | .10^*^ | -.05 | .01 | -.03 | -.04 | .07 |
| 6 | Civilian status (0 = other, 1 = married/partnership) |  |  |  |  |  | .44^**^ | .61^**^ | -.11^**^ | .09^*^ | -.05 | .11^*^ | -.09^*^ |
| 7 | Having children (0 = no, 1 = yes) |  |  |  |  |  |  | .21^**^ | -.19^**^ | .14^**^ | -.13^**^ | .15^**^ | -.19^**^ |
| 8 | Living situation |  |  |  |  |  |  |  | .00 | -.02 | .03 | .06 | .01 |
| 9 | MS-type (0 = PMS, 1 = RRMS) |  |  |  |  |  |  |  |  | -.37^**^ | .32^**^ | -.34^**^ | .52^**^ |
| 10 | Time since MS diagnosis (per 1 year increase) |  |  |  |  |  |  |  |  |  | -.25^**^ | .16^**^ | -.31^**^ |
| 11 | Current disease modifying treatment (0 = no, 1 = yes) |  |  |  |  |  |  |  |  |  |  | -.10^*^ | .17^**^ |
| 12 | Sum score MS symptoms |  |  |  |  |  |  |  |  |  |  |  | -.67^**^ |
| 13 | HRQoL |  |  |  |  |  |  |  |  |  |  |  |  |
|  |  |  |  |  |  |  |  |  |  |  |  |  |  |
|  | * p < .05 , ** p < .01 (one-tailed) | | | | |  |  |  |  |  |  |  |  |
|  |  |  |  |  |  |  |  |  |  |  |  |  |  |
|  | Correlations with job positions were calculated with the Spearman correlation coefficient. All other variables were calculated with the Pearson correlation coefficient. | | | | | | | | | | | | |
|  |  |  |  |  |  |  |  |  |  |  |  |  |  |

Abbreviations:

MS = Multiple Sclerosis

RRMS = Relapsing-remitting MS

PMS = Progressive MS

HRQoL: Health-related quality of life
